# Exploring HIV-related stigma and its impact on ethnic Mizo people living with HIV in Mizoram, Northeast India: a prospective observational study

**DOI:** 10.1186/s12981-025-00822-9

**Published:** 2025-12-07

**Authors:** Irene Lalhruaimawii, S. Sangthang, Danturulu Muralidhar Varma, U. K. Chandrashekar, Richard L. Chawngthu, Radhakrishnan Rajesh

**Affiliations:** 1 Department of Pharmacy Practice, Manipal College of Pharmaceutical Sciences, Manipal Academy of Higher Education, Manipal, 576 104 Karnataka India; 2 Department of Microbiology, Zoram Medical College and Hospital, Falkawn, Mizoram, 796005 India; 3 Department of Infectious Diseases, Kasturba Medical College, Manipal, Manipal Academy of Higher Education, Manipal, 576 104 Karnataka India; 4 Department of Medicine, Kasturba Medical College, Manipal, Manipal Academy of Higher Education, Manipal, 576 104 Karnataka India; 5 Care, Support and Treatment Division, Mizoram State AIDS Control Society, Aizawl, Mizoram, 796 001 India

**Keywords:** HIV stigma, PLHIV, ART, Adherence, Mizoram, India

## Abstract

**Background:**

In India, despite significant advancements in Antiretroviral Therapy (ART), stigma and discrimination remain major barriers for people living with HIV (PLHIV), often hindering ART adherence and compromising treatment outcomes. This study aimed to assess the determinants and contributing factors of HIV-related stigma among PLHIV in Mizoram, a northeastern state with one of the highest HIV prevalence rates in the country.

**Methods:**

A cross-sectional study was conducted among 300 PLHIV attending the ART Center, in Aizawl, Mizoram. Descriptive statistics, Chi-square tests, and binary logistic regression were used to assess factors associated with stigma and treatment adherence.

**Results:**

A total of 300 PLHIV were enrolled in the study, comprising 176 (58.7%) males and 124 (41.3%) females. In the internalized stigma domain, males experienced significantly higher stigma compared to females (aOR = 2.394, CI = 1.294–4.426, *p* = 0.005). In the felt-normative stigma domain, participants aged 41–50 years reported higher stigma levels compared to aged 51 years and above (aOR = 0.329, CI = 0.110–0.985, *p* = 0.047). Regarding medication adherence, 208 (69.3%) participants demonstrated optimal adherence to ART, while 92 (30.7%) had sub-optimal adherence.

**Conclusion:**

Most PLHIV in our study reported low to moderate stigma across domains. To reduce HIV related stigma among PLHIV, one should prioritize patient centric counselling, educational interventions in the form of mass communication, printed media etc., to ensure their psychological well-being and to create educational awareness involving the community and healthcare professionals to promote more positive thoughts on HIV which will reduce HIV related stigma in the society.

## Introduction

Worldwide, Human Immunodeficiency Virus (HIV) remains one of the persistent global pandemics, with the number of people living with HIV (PLHIV) continuing to rise [1]. In India, the first case of HIV was identified in 1986 from Chennai, Tamil Nadu [2]. Mizoram recorded its first case in 1990 among Injecting Drug Users (IDUs) [3]. Currently, there are approximately 2.4 million PLHIV in India with a prevalence rate of 0.21% [4]. In Mizoram, the estimated HIV prevalence is 2.7%, and at March 2025, the number of HIV-positive individuals detected in the state was 32, 544. Although this number may be comparatively lower than in other states, it has not seen a significant reduction over time [5, 6]. 

HIV is primarily transmitted among individuals who engage in high-risk sexual behaviors and among IDUs [7]. Due to this, it is often labelled as a disease associated with behaviors that are deemed perverted and sinful [8]. The resulting misconceptions and fear surrounding HIV contribute to negative attitudes and further cause stigmatization towards PLHIV [9]. With the advancement of Antiretroviral Therapy (ART) in India, HIV has evolved from a fatal disease into a chronic and manageable condition. However, stigma and discrimination continue to serve as major barriers for accessing effective prevention and treatment services for PLHIV [10]. 

According to the World Health Organization (WHO), stigma is defined as “a mark of shame, disgrace or disapproval which results in an individual being rejected, discriminated against, and excluded from participating in various areas of society” [11]. PLHIV often experiences stigma and discrimination from their families and communities, which not only exacerbates their medical condition but also transforms the issue into a social problem [12]. HIV related stigma does not merely hinder prevention, treatment, and control of new HIV infection, but also negatively affects the physical, social, and emotional well-being of those affected [13]. 

It is essential to identify why HIV is more stigmatized than other diseases. First, HIV is often perceived as the bearer’s behavior since the primary modes of transmission, such as unprotected sex and needle sharing, are considered voluntary and preventable. Second, greater stigma is associated with the perception of HIV as a terminal illness. Third, transmissible diseases are generally assumed to carry higher stigma. Finally, an illness affecting a person’s physical appearance and strength tends to be more stigmatized [14]. 

Although stigma is shaped by societal factors, it is crucial to recognize the origins and determinants of this stigma as they are deeply intertwined with cultural beliefs, ethics, and morals [7]. The individuals experiencing HIV related stigma can vary from person to person, and it can be emphasized in different ways. The first component can be termed as “enacted stigma,” where acts of discrimination and hostility are directly towards the person due to their stigmatized status. Hearing other's experiences of enacted stigma feels real even for those who have not faced discrimination themselves. The indirect way of learning enacted stigma refers to the second component, “vicarious stigma”. The third component, “felt-normative stigma,” refers to an individual’s awareness or belief that stigmatizing attitudes are common or widely accepted within the local community. The fourth component, “internalized stigma,” refers to the extent to which a person accepts stigma as true, or they see themselves in line with the negative view of others and accept it. Gaining a clear understanding of the specific nature of stigma is essential for designing effective interventions to promote health among PLHIV [15]. While several studies in India have explored HIV-related stigma, there is limited research on its prevalence and the negative impact it has on PLHIV in the Northeastern region, particularly in Mizoram. Apart from one study that focused exclusively on HIV internalized stigma, little is known about the broader experience of stigma among PLHIV in this region [3]. Therefore, this study aims to assess HIV related stigma and its contributing factors, which may prevail among PLHIV in Mizoram, in the Northeastern part of India.

## Methodology

The study was conducted at the ART Center, Kulikawn Hospital, Aizawl, Mizoram, which serves over 3000 alive registered PLHIV. The center records an increase of approximately 30 or more inclusive of trans-in from other ART centers and newly enrolled PLHIV clients each month. The participants were recruited within a time frame between January 2023 and May 2024.

The study included adult PLHIV aged 18 years or older who had been on ART for at least one month, and were receiving services at the ART center, Kulikawn, Aizawl. Information about the study was explained to the participants before obtaining a handwritten Informed Consent. Participants were informed of their right to refuse or discontinue participation at any time. Privacy was strictly maintained by conducting interviews at a private cabin, and confidentiality regarding personal identifiers such as ART registration numbers was ensured by excluding them from the survey questionnaires.

The HIV Stigma Scale questionnaire (HIV-SSQ) developed by Steward *et al.,* was used to assess HIV related stigma, with permission obtained prior to its use [15]. The original English version of the questionnaire was translated into the local language i.e., Mizo, to ensure comprehension. The Visual Analogue Scale (VAS) was also used to assess ART adherence. Social demographic details, including gender, age, education qualification, employment status, marital status, residence, CD4 cell count, and Viral load, were recorded for all participants during recruitment. Patients who were unwilling or unable to sign Informed Consent, visually impaired, and hearing loss were excluded from the study.

### HIV- Stigma Scale Questionnaire (HIV-SSQ)

Stigma was assessed using the 40-item HIV-SSQ [15], which was broadly classified into four domains i.e., enacted stigma (10 items), vicarious stigma (10 items), felt-normative stigma (10 items), and internalized stigma (10 items). The enacted stigma score can range from 0 to 10, with responses coded as No = 0 and Yes = 1 for each question. Scores for vicarious stigma, felt-normative stigma, and internalized stigma scores can range from 0 to 30 in each domain, respectively. The total scores can range from 0 to 100, with higher scores indicative of greater levels of stigma. For this study, overall stigma and subscale scores were categorized into two groups: low or moderate stigma, and high stigma, using the 33rd and 66th percentile cut-off values from the distribution of scores. This categorization was exclusively done for this study.

### Visual analogue scale (VAS)

 The VAS was used to assess ART adherence. Participants were shown numbers ranging from 0 to 100 and asked to indicate the number that best represents the percentage of pills taken in the past month. For example, 0% meant that no drug was taken, 50% meant that half of the prescribed drug was taken, and 100% meant that every dose of the prescribed drug was taken. Participants with adherence rates of more than 95% were considered optimally adherent, while those with adherence rates of less than 95% were classified as sub-optimally adherent [16, 17]. 

### Statistical analysis

 The continuous variables were reported as means and standard deviations (SD). The Chi-Square test was used to analyze the association among different nominal variables. In addition to descriptive statistics and Chi-square tests, binary logistic regression analysis was used to estimate adjusted odds ratios (aOR) with 95% confidence intervals for factors associated with stigma and adherence. Variables showing a statistically significant difference in bivariate analysis were included in the multivariate logistic regression model. A *p-*value of less than 0.05 was considered statistically significant. Data analysis was performed using SPSS version 20 (IBM, USA).

### Ethical considerations

 Ethical approval was obtained from the Institutional Ethical Committee (IEC), Civil Hospital, Aizawl, Mizoram (Ref. No. 12018/1/13/-CH(A)/IEC/99) prior to the initiation of the study. Following Ethical approval, permission was obtained from the Mizoram State AIDS Control Society to recruit participants from the ART Center, Kulikawn Hospital, Aizawl, Mizoram, as they are the stakeholders involved in addressing the needs of the PLHIV community.

## Results

### Socio-demographic characteristics of study participants

A total of 300 participants were recruited in the study, including 176 (58.7%) male and 124 (41.3%) female PLHIV participants. The mean age was 35.03 (± 9.002 SD) years, with ages ranging from 18 to 68 years. Most participants were married (127, 42.3%), followed by unmarried (96, 32%), and widowed or divorced (77, 25.7%) individuals. A large majority (85%) of the participants resided in urban areas, and most (67.7%) were employed. Regarding education, 173 (57.7%) participants had completed below 10th grade, while 127, (42.3%) had attained a higher secondary level or its equivalent degree. In terms of viral load, most participants,175 (74.4%) had a viral load below 150 copies per mL, while only 14 (6.0%) had a viral load above 1000 copies per mL. Additionally, more than half of the participants, 217 (72.8%) had a CD4 cell count between 201 and 600 per cubic millimeter, while 32 (10.7%) of them had a CD4 cell count below 200 per cubic millimeter. The mean adherence level among the study subjects was 94.80 (± 9.2 SD), and 132 (44%) of the participants had been on ART for more than 36 months. A total of 208 (69.3%) participants demonstrate optimal adherence, while 92 (30.7%) exhibited sub-optimal adherence (Table 1).

**Table 1:** Socio-demographic characteristics of study participants

Characteristics	Group	Total (*N* = 300)
Gender	Male	176 (58.7%)
	Female	124 (41.3%)
Age (group)	18 to 30 years	102 (34%)
	31 to 40 years	120 (40%)
	41 to 50 years	57 (19%)
	51 years and above	21 (7%)
Marital status	Unmarried	96 (32%)
	Married	127 (42.3%)
	Widowed	29 (9.7%)
	Divorce	48 (16%)
Residence	Rural	45 (15%)
	Urban	255 (85%)
Employment status	Unemployed	97 (32.3%)
	Employed	203 (67.7%)
Education level	10 standard and below	173 (57.7%)
	Diploma and 12 standard	84 (28.0%)
	Graduate and above	43 (14.3%)
Duration of ART drugs	Below 12 months	93 (31%)
	Between 13 and 36 months	75 (25%)
	Above 36 months	132 (44%)
Viral load level	Below 100	175 (74.4%)
	101 to 1000	46 (19.6%)
	Above 1000	14 (6.0%)
CD4 count level	200 and below	32 (10.7%)
	between 201 and 600	217 (72.8%)
	601 and above	49 (16.5%)
ART adherence level	Sub-optimal adherence	92 (30.7%)
	Optimal adherence	208 (69.3%)

Data are presented as counts (percentages)

### Assessment of stigma level among PLHIV

**Fig. 1:**
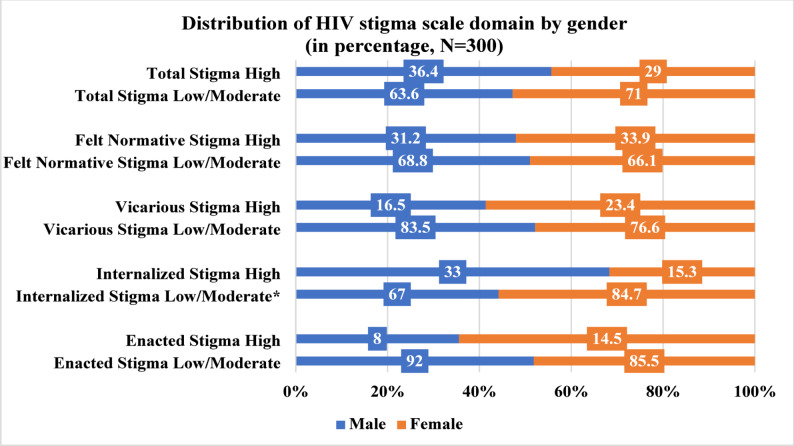
Distribution of HIV stigma scale domain by gender (* The p-value is less than 0.05)

The overall mean stigma score in our study is 17.49 (± 10.27 SD). The mean scores for each of the stigma sub domain scale is 0.13 (± 0.43 SD, possible range 0–10) for enacted stigma, 2.33 (± 2.44 SD, possible range 0–30) for vicarious stigma, 9.78 (± 6.66 SD, possible range 0–30) for felt-normative stigma and 5.26 (± 4.56 SD, possible range 0–30) for internalized stigma respectively. The prevalence of high stigma is 29.0% among the study population. Among each stigma domain, high internalized stigma was found to be higher among male participants (33%) than female participants (15.3%) as in Fig. 1.

The total stigma score and its association with socio‑economic characteristics of study participants were analyzed using the Chi-Square test. The Stigma level was not significantly different between the employed and non-employed participants. However, it was observed that there were higher counts of low or moderate stigma than high stigma levels within the group. Additionally, there was more of a lower or moderate stigma level in both rural and urban residences though this difference was not statistically significant. The prevalence of stigma levels among different socio-economic variables is also displayed (Table 2).

**Table 2:** Socio-economic characteristics and total stigma level

Characteristics	Group	Low or moderate	High	Total (*N* = 300)	*p*-value
Age (group)	18 to 30 years	59 (19.7%)	43 (14.3%)	102 (34.0%)	0.055
	31 to 40 years	82 (27.3%)	38 (12.7%)	120 (40.0%)	
	41 to 50 years	45 (15.0%)	12 (4.0%)	57 (19.0%)	
	51 years and above	14 (4.7%)	7 (2.3%)	21 (7.0%)	
Gender	Male	112 (37.3%)	64 (21.3%)	176 (58.7%)	0.214
	Female	88 (29.3%)	36 (12.0%)	124 (41.3%)	
Marital status	Single	56 (18.7%)	40 (13.3%)	96 (32.0%)	0.130
	Married	87 (29.0%)	40 (13.3%)	127 (42.3%)	
	Widowed	23 (7.7%)	6 (2.0%)	29 (9.7%)	
	Divorce	34 (11.3%)	14 (4.7%)	48 (16.0%)	
Employment	Unemployed	70 (23.3%)	27 (9.0%)	97 (32.3%)	0.191
	Employed	130 (43.3%)	73 (24.3%)	203 (67.7%)	
Residence	Rural	28 (9.3%)	17 (5.7%)	45 (15.0%)	0.497
	Urban	172 (57.3%)	83 (27.7%)	255 (85%)	
Education level	10 standard and below	124 (41.3%)	49 (16.3%)	173 (57.7%)	0.055
	Diploma and 12 standards	53 (17.7%)	31(10.3%)	84 (28.0%)	
	Graduate and above	23 (7.7%)	20 (6.7%)	43 (14.3%)	
Duration of ART drugs	Below 12 months	59 (19.7%)	34 (11.3%)	93 (31.0%)	0.141
	Between 13 and 36 months	57 (19.0%)	18 (6.0%)	75 (25.0%)	
	Above 36 months	84 (28.0%)	48 (16.0%)	132 (44.0%)	
CD4 count level	200 and below	21 (7.0%)	11 (3.7%)	32 (10.7%)	0.521
	Between 201 and 600	141 (47.3%)	76 (25.5%)	217 (72.8%)	
	601 and above	36 (12.1%)	13 (4.4%)	49 (16.4%)	
ART adherence level	Sub-optimal adherence	63 (21.0%)	29 (9.7%)	92 (30.7%)	0.692
	Optimal adherence	137 (45.7%)	71 (23.6%)	208 (69.3%)	

Data are presented as counts (percentages)

### Assessment of sub-stigma scale among PLHIV

The study found that there was a statistically significant difference in the stigma level for the variables of “age group”, “gender”, “marital status”, and “duration of ART taken” in the subscale of internalized stigma (i.e., *p*-value of 0.039; 0.001; 0.010 and 0.011). It was also observed that the subscale stigma level of felt-normative stigma was significantly different within the variables “age group” and “education level” of the participants (i.e., *p*-value of 0.021; 0.044). In addition, the vicarious stigma level was statistically different among the employed and unemployed (*p* = 0.019) PLHIV participants, while none of the variables were found to have a significant difference for the enacted stigma level. The HIV stigma subscales i.e., internalized stigma, felt-normative stigma, vicarious stigma, and enacted stigma, and their association with the socio-demographic characteristics are described (Table 3).

**Table 3:** Socio-economic characteristics and sub-stigma level

Characteristics	Group	Internalized stigma level			Felt-normative stigma level			Vicarious stigma level			Enacted stigma level		
		Low or moderate	High	*p* -value	Low or moderate	High	*p ****-***value	Low or moderate	High	*p* ***-***value	Low or moderate	High	*p* ***-***value
Age (group)	18 to 30 yrs	67 (22.3%)	35 (11.7%)	0.039^*^	60 (20%)	42 (14%)	0.021^*^	85 (28.3%)	17 (5.7%)	0.736	89 (29.7%)	13 (4.3%)	0.859
	31 to 40 yrs	90 (30%)	30 (10%)		85 (28.3%)	35 (11.7%)		94 (31.3%)	26 (8.7%)		108 (36%)	12 (4%)	
	41 to 50 yrs	48 (16%)	9 (3%)		46 (15.3%)	11 (3.7%)		47 (15.7%)	10 (3.3%)		52 (17.3%)	5 (1.7%)	
	> 51 years	18 (6%)	3 (1%)		12 (4%)	9 (3%)		16 (5.3%)	5 (1.7%)		19 (6.3%)	2 (0.7%)	
Gender	Male	118 (39.3%)	58 (19.3%)	0.001^*^	121 (40.3%)	55 (18.3%)	0.707	147 (49.0%)	29 (9.7%)	0.141	162 (54.0%)	14 (4.7%)	0.087
	Female	105 (35%)	19 (6.3%)		82 (27.3%)	42 (14.0%)		95 (31.7%)	29 (9.7%)		106 (35.3%)	18 (6.0%)	
Marital status	Single	61 (20.3%)	35 (11.7)	0.010^*^	57 (19.0%)	39 (13.0%)	0.145	84 (28.0%)	12 (4.0%)	0.101	89 (29.7%)	7 (2.3%)	0.088
	Married	101 (33.7%)	26 (8.7%)		94 (31.3%)	33 (11.0%)		100 (33.3%)	27 (9.0%)		115 (38.3%)	12 (4.0%)	
	Widowed	26 (8.7%)	3 (1%)		20 (6.7%)	9 (3.0%)		24 (8.0%)	5 (1.7%)		26 (8.7%)	3 (1.0%)	
	Divorce	35 (11.7%)	13 (4.3%)		32 (10.7%)	16 (5.3%)		34 (11.3%)	14 (4.7%)		38 (12.7%)	10 (3.3%)	
Employment	Unemployed	73 (24.3%)	24 (8%)	0.888	66 (22%)	31 (10.3%)	1.00	86 (28.7%)	11 (3.7%)	0.019^*^	86 (28.7%)	11 (3.7%)	0.842
	Employed	150 (50%)	53 (17.7%)		137 (45.7%)	66 (22%)		156 (52.0%)	47 (15.7%)		182 (60.7%)	21 (7.0%)	
Residence	Rural	33 (11%)	12 (4%)	0.855	30 (10.0%)	15 (5.0%)	0.864	34 (11.3%)	11 (3.7%)	0.412	41 (13.7%)	4 (1.3%)	0.799
	Urban	190 (63.3%)	65 (21.7%)		173 (57.7%)	82 (27.3%)		208 (69.3%)	47 (15.7%)		227 (75.7%)	28 (9.3%)	
Education level	10 standard and below	137 (45.7%)	36 (12%)	0.058	122 (40.7%)	51 (17%)	0.044^*^	146 (48.7%)	27 (9.0%)	0.158	160 (53.3%)	13 (4.3%)	0.081
	Diploma and 12 standards	55 (18.3%)	29 (9.7%)		59 (19.7%)	25 (8.3%)		64 (21.3%)	20 (6.7%)		70 (23.3%)	14 (4.7%)	
	Graduate and above	31 (10.3%)	12 (4%)		22 (7.3%)	21 (7%)		32 (10.7%)	11 (3.7%)		38 (12.7%)	5 (1.7%)	
Duration of ART drugs	< 12 months	59 (19.7%)	34 (11.3%)	0.011^*^	61 (20.3%)	32 (10.7%)	0.642	76 (25.3%)	17 (5.7%)	0.352	83 (27.7%)	10 (3.3%)	0.653
	13 to 36 months	62 (20.7%)	13 (4.3%)		54 (18%)	21 (7%)		64 (21.3%)	11 (3.7%)		69 (23%)	6 (2%)	
	> 36 months	102 (34%)	30 (10%)		88 (29.3%)	44 (14.7%)		102 (34.0%)	30 (10%)		116 (38.7%)	16 (5.3%)	
CD4 count level	< 200	25 (8.4%)	7 (2.3%)	0.181	22 (7.4%)	10 (3.4%)	0.585	25 (8.4%)	7 (2.3%)	0.200	31 (10.4%)	1 (0.3%)	0.231
	201 and 600	155 (52%)	62 (20.8%)		143 (48%)	74 (24.8%)		171 (57.4%)	46 (15.4%)		190 (63.8%)	27 (9.1%)	
	> 601	41 (13.8%)	8 (2.7%)		36 (12.1%)	13 (4.4%)		44 (14.8%)	5 (1.7%)		45 (15.1%)	4 (1.3%)	
ART adherence level	Sub Optimal Adherence	67 (22.3%)	25 (8.4%)	0.774	67 (22.3%)	25 (8.3%)	0.230	73 (24.3%)	19 (6.4%)	0.752	81 (27.0%)	11 (3.7%)	0.686
	Optimal Adherence	156 (52%)	52 (17.3%)		136 (45.3%)	72 (24%)		169 (56.3%)	39 (13.0%)		187 (62.3%)	21 (7.0%)	

Data are presented as counts (percentages)

* Statistically significant

The independent association between socio-demographic variables and HIV stigma subscales was analyzed using logistic regression (Table 4). It was observed that in the internalized stigma domain, male participants (aOR = 2.394, CI = 1.294–4.426, *p* = 0.005) experienced more stigma than female participants. Meanwhile, in felt-normative stigma domain, individuals aged 41–50 years (aOR = 0.329, CI = 0.110–0.985, *p* = 0.047) experienced more stigma as compared to those 51 years and above. Similarly, we observe that study participants who are unemployed (aOR = 0.425, CI = 0.209–0.861, *p* = 0.018) experience more stigma than employed in the vicarious stigma domain. However, the correlation within stigma subscales along with the overall stigma scale, only the correlation between internalized stigma was significantly related to enacted stigma (*p* < 0.01).

**Table 4:** Logistic regression between socio-economic characteristics and sub-stigma level

Characteristics	Group	Internalized stigma level			Felt-normative stigma level		Vicarious stigma level	
		Prevalence	aOR (95% CI)	*p-value*	aOR (95% CI)	*p-value*	aOR (95% CI)	*p-value*
Age (group)	18 to 30 yrs	102 (34%)	1.744 (0.426–7.147)	0.439	0.915 (0.345–2.430)	0.895	-	
	31 to 40 yrs	120 (40%)	1.383 (0.353–5.411)	0.641	0.550 (0.205–1.475)	0.235	-	
	41 to 50 yrs	57 (19%)	0.953 (0.221–4.104)	0.948	0.329 (0.110–0.985)	0.047^*^	-	
	> 51 years	21 (7%)	Reference	0.588	Reference	0.037^*^	-	
Gender	Male	176 (58.7%)	2.394 (1.294–4.426)	0.005^*^	-		-	
	Female	124 (41.3%)	Reference		-		-	
Marital status	Single	96 (32%)	0.974 (0.424–2.238)	0.951	-		-	
	Married	127 (42.3%)	0.640 (0.286–1.432)	0.278	-		-	
	Widowed	29 (9.7%)	0.447 (0.107–1.869)	0.270	-		-	
	Divorce	48 (16%)	Reference	0.444	-		-	
Employment	Unemployed	97 (32.3%)			-		0.425 (0.209–0.861)	0.018^*^
	Employed	203 (67.7%)			-		Reference	
Education level	10 standard and below	173 (57.7%)			0.473 (0.235–0.950)	0.035^*^	-	
	Diploma and 12 standards	84 (28.0%)			0.460 (0.212–0.998)	0.049^*^	-	
	Graduate and above	43 (14.3%)			Reference	0.082	-	
Duration of ART drugs	< 12 months	93 (31%)	1.692 (0.897–3.192)	0.104	-		-	
	13 to 36 months	75 (25%)	0.679 (0.321–1.437)	0.312	-		-	
	> 36 months	132 (44%)	Reference	0.050	-		-	

Data are presented as counts (percentages)

* Statistically significant

*aOR* Adjusted Odds Ratio; *CI* Confidence Interval

## Discussion

The determinants of HIV stigma are complex, diverse, and continue to pose a challenge in the fight against the HIV epidemic. In the current context, HIV-related stigma is understood as a social process that can be addressed through social actions, such as support from families, communities, and healthcare facilities [18, 19]. Although Northeast India has the highest prevalence of HIV, and there is a pressing need to address the stigma burden, there is a scarcity of community-based studies on PLHIV that assess the prevalence of stigma and its associated factors, especially in Mizoram. This gap highlights the importance of our study, which plays a crucial role in addressing the key factors contributing to HIV stigma in Mizoram, as compared to the existing literature.

Many of the PLHIV registered at the ART center were unable to participate in the study due to the high number of caretakers visiting the center on behalf of PLHIV currently on ART. This observation indicates that PLHIV registered at this center receive strong support from their families. In our study, it was observed that HIV-related stigma plays a significant role among PLHIV in Mizoram, particularly within the Mizo communities, as most of the recruited participants were locals who spoke the regional language, Mizo.

A significantly greater number of participants in this study reported low or moderate internalized stigma, a finding similar to that of the pilot study by Gohain *et al.;*(2014), which was conducted in the same geographical region. Their study found that 64.5% had moderate internalized stigma, 35.5% had low internalized stigma, and none had high internalized stigma. This finding can be attributed to the fact that the Mizo community is a close-knit society with no class distinctions, and the whole community is involved in most of the social events, particularly the birth of a child, marriage, and the death of a person. The majority of the Mizo people identify as Christians, and their social life and worldview are shaped and influenced by the teachings and structures of the Christian Church, which strongly reflects the current situation of internalized stigma among PLHIV in Mizoram [3]. In contrast, a study conducted by Thomas *et al.,* (2005) in Chennai and Subramanian *et al.,* (2009) in Bangalore reported much higher levels of internalized stigma, with 63% and 83% of participants, respectively, experiencing high levels of internalized stigma [20, 21]. 

The excerpt from the research paper discusses gender differences in internalized stigma related to HIV status. The main finding is that male participants had significantly higher internalized stigma scores compared to female participants, with the mean score for men being 6.31 (± 4.36 SD) and for women 3.37 (± 4.44 SD), with a *p*-value less than 0.05, which is comparable with the study in Haryana by Sahoo *et al.,* (2020) [10]. The authors suggest that this may be due to men being more likely to experience stigma in the workplace, as many of them are wage earners and have large public and professional circles, which can be attributed to our current findings [22]. Stigma could also be linked to guilt over transmitting HIV to their spouses that restrain them from disclosing their identity and bridging the gap between their relationship, even though our study is unable to measure it exclusively. Similar findings were also observed in other studies [23–25]. However, the study also acknowledges findings from other research that suggest women may experience higher stigma. In India, women may have limited control over condom use within relationships, and attempts to negotiate its use can sometimes be perceives as a sign of infidelity or irresponsibility, leading to stigma, neglect, or even violence. Factors, such as the societal pressures to conceive, domestic violence, and women’s lower societal status may further contribute to higher internalized stigma among women [8, 20, 26–28]. While men generally experience more stigma in our study, gender-based differences in stigma are complex, and factors like societal roles, relationship dynamics, and gender inequality influence how stigma is internalized.

This part of the paper highlights that no significant differences were found in total stigma levels (categorized as Low/Moderate vs. High) when analyzed across various demographic variables, as presented in Table 2. The study's findings align with those of local researchers and a report by Lee *et al.,* (2002) from the United States [29]. However, the paper contrasts these findings with a study by Sushila *et al.,* (2022) in Nepal, which did find significant differences in stigma levels based on demographic factors such as gender, marital status, education, and occupation [30]. 

This section of the study reports that higher internalized stigma levels were more prevalent among younger individuals (18–30 years) compared to the higher age-group [10]. The findings are in contrast with those of Md. Tanvir *et al.,* (2012) and Barbara *et al.,* (2011), claim that internalized stigma tends to increase with age [24, 27]. The researchers suggest that younger individuals may be more vulnerable to internalized stigma due to engaging in risky behaviors, such as unsafe sexual practices or using injectable drugs, which can lead to self-blame and poor adherence to ART. Therefore, it is a future directive to look into and investigate the actual scenario of PLHIV in Mizoram. Further, unmarried participants, and those who had been on ART for less than 12 months, experienced internalized stigma with reference to the other group as seen in Table 4. It could be due to the unavailability of spouses or partners to support each other among unmarried participants, and there is a need for professional support to promote satisfaction with ART services among PLHIV. Additionally, participants aged 18–30 years and those with an education level of 10th grade or below were more likely to experience high felt-normative stigma that requires further exploration. The study also observed that employed individuals with HIV had higher levels of vicarious stigma compared to unemployed individuals, potentially because disclosing their HIV status might lead to curiosity and discrimination from their co-worker [22]. 

The study also employs logistic regression to analyze the odds of stigma sub-scales across various parameters, which are detailed in Table 4. The analysis reveals that the odds of internalized stigma for male participants are 2.394 times higher compared to female participants. However, there were no significant differences observed in internalized stigma when considering age group, marital status, or duration of ART. In terms of felt-normative stigma, participants aged 41–50 years had odds of 0.329 when compared to those aged over 51 years, indicating lower odds of felt-normative stigma in this age group. However, there was no statistically significant difference based on educational level, which contrasts with the findings presented in Table 3. Meanwhile, the analysis found that unemployed participants had odds of 0.018 for experiencing vicarious stigma compared to employed participants, suggesting that employed participants were more likely to experience vicarious stigma, possibly due to workplace dynamics related to ART use.

Furthermore, the study examines the correlation between internalized stigma and enacted stigma as observed in the current research. This finding aligns with a report from Bangalore, India by Steward *et al.,* (2012), which suggests that internalized stigma can delay individuals from seeking care after being diagnosed with HIV. The correlation between these two types of stigmas highlights that the stigma people internalize can directly influence how they perceive and experience enacted stigma in their communities. This correlation presents an important area for intervention. By targeting enacted stigma, through awareness programs or other interventions, it may be possible to reduce the negative effects of both internalized and enacted stigma on PLHIV [31]. 

## Conclusion

In conclusion, the authors acknowledge the study’s limitations but emphasize its potential implications for improving the health and quality of life of PLHIV in Mizoram. Although many participants reported experiencing low or moderate levels of stigma, the authors highlight the need of developing community-based stigma reduction programs in Mizoram, strengthening patient-centric counselling and peer support at ART centers, integrating gender-sensitive interventions since males reported higher stigma. The authors also advocate for education and awareness campaigns tailored to tribal populations and for implementing HIV stigma-reduction strategies through educational intervention trials that utilize mass communication, print media, and social media platforms, where information can be disseminated rapidly and effectively. The authors further recommend adopting multi-dimensional intervention approaches involving communities, healthcare practitioners, and social workers to address social stigma. Future research should include longitudinal studies on stigma and treatment adherence, qualitative explorations of the lived experiences of PLHIV, and intervention trials evaluating the effectiveness of stigma-reduction programs. Additionally, efforts to raise awareness and building supportive environments for HIV affected communities are crucial to mitigating HIV-related stigma in the society.

## Study limitation

The study acknowledges several limitations that may affect the generalizability and accuracy of the findings. The participants were recruited from HIV treatment settings, meaning the findings may not apply to other groups of PLHIV who are not in similar healthcare settings, limiting the broader applicability of the results. Approximately 30% of PLHIV were not included in the study because of time constraints related to their visits to the ART center. Since stigma levels were self-reported by the participants, there is a risk of response bias, where participants may either underreport or overreport their levels of stigma due to social desirability or fear of judgment.

## Data Availability

Data will be available only upon request to the corresponding author.

## Abbreviations

ART: Antiretroviral Therapy
PLHIV: People Living with HIV
HIV: Human Immunodeficiency Virus
IDU: Injecting Drug Users
WHO: World Health Organization
VAS: Visual Analog Scale
IEC: Institutional Ethics Committee
SD: Standard Deviation
HIV-SSQ: HIV Stigma Scale Questionnaire
